# Nanodrugs for Subcutaneous Mycoses: Applications, Antifungal Performance, and Translational Perspectives

**DOI:** 10.3390/microorganisms14010187

**Published:** 2026-01-14

**Authors:** Micaela Federizzi, Eduarda Canosa Adegas, Alexandre Meneghello Fuentefria, Stefanie Bressan Waller

**Affiliations:** 1 Graduate Program in Pharmaceutical Sciences, Federal University of Rio Grande do Sul, Porto Alegre 90610-000, RS, Brazil; federizzi.mica@gmail.com (M.F.); alexandre.fuentefria@ufrgs.br (A.M.F.); 2 Applied Mycology Research Laboratory, Federal University of Rio Grande do Sul, Porto Alegre 90620-170, RS, Brazil; vetdudadegas@gmail.com; 3 Graduate Program in Agricultural and Environmental Microbiology, Federal University of Rio Grande do Sul, Porto Alegre 90035-003, RS, Brazil; 4 Laboratory of Veterinary Mycology, Faculty of Veterinary, Federal University of Rio Grande do Sul, Porto Alegre 91540-000, RS, Brazil

**Keywords:** nanotechnology, subcutaneous mycoses, antifungal nanosystems

## Abstract

Subcutaneous mycoses are a heterogeneous group of chronic fungal infections, usually acquired through traumatic inoculation of environmental fungi and particularly severe in immunocompromised and critically ill patients. These infections involve pathogens with marked morphological and physiopathological diversity, resulting in significant diagnostic and therapeutic challenges. Conventional treatment relies on systemic antifungals such as amphotericin B, itraconazole, and other azoles; however, these therapies are often limited by poor tissue penetration, adverse effects, and prolonged treatment regimens, especially in vulnerable patient populations. In this context, nanodrugs have emerged as promising alternatives by improving solubility, stability, bioavailability, and targeted delivery to infection sites. This review conducted a comprehensive literature search in PubMed, SciELO, ScienceDirect, Web of Science, and Scopus, identifying 31 eligible studies that developed and evaluated antifungal nanosystems using *in vitro*, *ex vivo*, and/or *in vivo* models. Quantitative outcomes included minimum inhibitory concentration (MIC), colony-forming units (CFU), inhibition halo diameter, and survival assays. Overall, the evidence indicates that several nanosystems may overcome key pharmacological limitations of conventional antifungals and enhance therapeutic outcomes. Nevertheless, important translational challenges remain, including toxicity, long-term safety, scalability, and regulatory approval, which must be addressed before clinical implementation.

## 1. Introduction

Subcutaneous mycoses constitute a heterogeneous group of chronic fungal infections, frequently acquired through traumatic inoculation of environmental agents present in soil, vegetation, or decomposing organic matter [1,2]. Several fungal genera can affect subcutaneous tissue, including *Sporothrix* spp., *Rhizopus* spp., *Mucor* spp., *Lichtheimia* spp., *Syncephalastrum* spp., *Cladosporium* spp., *Cladophialophora* spp., *Bipolaris* spp., and *Paracoccidioides* spp., which exhibit wide morphological and physiopathological diversity, posing significant therapeutic challenges [3,4]. Although subcutaneous infections caused by *Candida* species are rare, they have been reported, particularly in immunocompromised patients, and should be considered in the spectrum of subcutaneous mycoses [5].

In classical subcutaneous mycoses, such as sporotrichosis, phaeohyphomycosis, and hyalohyphomycosis, infection occurs through traumatic inoculation of the skin and subcutaneous tissue, with possible lymphatic dissemination frequently affecting otherwise healthy individuals in endemic areas [6,7]. Other subcutaneous mycoses include mucormycosis and classical paracoccidioidomycosis, which present aggressive and rapidly progressive courses particularly in hospitalized or immunocompromised patients [8,9]. Mucormycosis mainly affects immunocompromised patients, has gained relevance in critical care settings, and is associated with angiotropic invasion, tissue necrosis, and recent increases in incidence, particularly after the COVID-19 pandemic [10]. The diversity of clinical manifestations and the heterogeneity of the causative agents hinder early diagnosis and compromise therapeutic response [11].

The conventional treatment of these infections is based on systemic antifungals such as amphotericin B, itraconazole, voriconazole, posaconazole, and terbinafine [12,13]. Despite their proven efficacy, these therapies present several limitations, such as gastrointestinal effects, low tissue penetration, severe adverse events, drug interactions, and the need for prolonged treatment, which often results in fungal recurrences and resistance [12,14]. Moreover, the financial burden constitutes a significant obstacle, as many of these drugs require daily administration for long periods, ranging from 3 to 6 months and, in complex cases, extending beyond one year [15]. In contrast, nanostructured formulations may allow for reduced dosing frequency and shorter treatment durations, potentially lowering overall costs while maintaining therapeutic efficacy [16].

In this context, nanotechnology emerges as a promising strategy to circumvent the pharmacological limitations of conventional therapies, exemplified by nanodrugs (nanosystems capable of encapsulating, adsorbing, or carrying bioactive molecules), which enhance drug solubility, stability, and bioavailability, while also enabling controlled release and specific targeting to the site of infection [17,18]. These properties have driven growing interest in nanotechnology-based antifungal formulations, particularly due to their potential to improve therapeutic efficacy while reducing systemic toxicity.

Despite these advantages, most antifungal nanosystems remain at the preclinical stage, with limited clinical accessibility associated with high production costs, regulatory complexity, and challenges related to large-scale manufacturing. Consequently, their current use is largely restricted to experimental settings or specialized centers, and their prevalence in routine clinical practice remains low. From a translational perspective, continued efforts are required to bridge laboratory findings and clinical application. In this context, the present review systematically summarizes the most promising antifungal nanosystems investigated for subcutaneous fungal infections, highlighting the developed nanosystems, incorporated drugs, routes of administration, and experimental methodologies employed.

## 2. Materials and Methods

This review was conducted using a narrative and systematized approach. A comprehensive literature search was performed in the PubMed, SciELO, Science Direct, Web of Science, and Scopus databases, including only original publications made available between January 2015 and August 2025.

The search strategy was based on the use of English descriptors combined with Boolean operators (AND/OR), as detailed in Table S1. These terms were individually adapted for each database according to their specific features, enabling an efficient screening of titles, abstracts, and keywords.

The eligibility criteria were defined in two categories. For inclusion criteria, studies were selected if they: (a) described the development and characterization of nanosystems with antifungal activity; (b) evaluated their *in vitro, ex vivo*, and/or *in vivo* efficacy against fungi associated with subcutaneous mycoses; and (c) reported quantitative antifungal activity data, such as minimum inhibitory concentration (MIC), colony-forming units (CFU), minimum fungicidal concentration (MFC), inhibition zone diameter (IZD), or survival assays.

Regarding the exclusion criteria, the following were disregarded: (a) review articles, commentaries, or meta-analyses; (b) studies without experimental data; (c) duplicate publications across the consulted databases; and (d) works that did not involve nanosystems or lacked a direct relationship with subcutaneous fungal infections. Review articles were not included in the present analysis but provide important background on the evolution, challenges, and translational perspectives of antifungal nanosystems.

Following the initial screening of the selected databases, 661 records were identified. After duplicate removal, 399 studies proceeded to title and abstract assessment, during which 164 reviews, 32 case reports, and 100 book chapters were excluded. Subsequently, 103 articles remained for full-text evaluation. In this phase, 42 studies that did not address nanotechnology and 30 that did not involve the fungal genera of interest were excluded. Ultimately, 31 studies fully met the eligibility criteria and were included in the review.

## 3. Assessed Fungal Genera and Species

The analysis of the 31 selected studies demonstrates the wide diversity of pathogenic fungi employed as experimental models for evaluating the efficacy of nanosystems, encompassing both opportunistic species and classical agents of subcutaneous mycoses. The selection included representatives of the main clinical groups of medical relevance, including *Mucorales* spp., *Penicillium* spp., *Cryptococcus* spp., *Sporothrix* spp., and dematiaceous fungi associated with eumycetoma and chromoblastomycosis (Table 1).

Fungi of the order *Mucorales* were the most frequently evaluated, reflecting the increasing incidence and severity of mucormycosis, particularly in immunosuppressed patients and in cases associated with COVID-19. Species such as *Rhizopus arrhizus*, *R. microsporus*, *R. delemar*, *Mucor circinelloides*, *M. racemosus*, *M. indicus*, and *Lichtheimia corymbifera* were tested across multiple studies, using both clinical isolates and reference strains [19,20,21,22,23,24,25,26,27,28,29,30,31,32,33,34]. These species are characterized by their thick cell wall and high resistance to azole antifungals, features that justify their selection as primary targets for the development of innovative formulations [35].

Species of the genus *Penicillium*, such as *P. notatum*, *P. commune*, and *P. digitatum*, were included as models of rapidly growing filamentous fungi with complex cell walls, enabling the comparison of the nanocarriers’ spectrum of action against organisms with distinct structural characteristics and of environmental and food-related relevance [20,25,26,27,29,36,37]. In addition, *Cryptococcus neoformans* was evaluated in both *in vitro* and murine models, standing out due to its polysaccharide capsule and its clinical relevance in infections of the central nervous system [5,38,39].

In sporothrichosis, the genus *Sporothrix* was extensively investigated due to its clinical and epidemiological importance in humans [40] and animals [41] species such as *S. brasiliensis*, *S. schenckii*, and *S. globosa* were tested using different reference strains and clinical isolates, including zoonotic variants obtained from felines [5,38,41,42,43,44,45,46,47,48,49]. Traits such as thermotolerance, melanin production, and immune-evasion capacity were relevant for understanding the response to the nanostructured formulations [44,48].

**Table 1:** Antifungal efficacy of different nanosystems against etiological agents of subcutaneous mycoses.

Fungal Genus/Species	Nanosystems	In Vitro/In Vivo/Ex Vivo Studies	In Vitro Antifungal Activity Assays	Main Antifungal Results	Ref
*Candida albicans*, *Mucor indicus*, *Aspergillus flavus*, *A. fumigatus*, *A. niger*, *Penicillium notatum*	Nitrogen-doped carbon quantum dots (N/CQDs) and nitrogen-doped mesoporous carbon (N/MC)	*In vitro* *In vivo*-rat	MIC, IZD, MFC, percentage of inhibition	N/CQDs and N/MC inhibited Mucor by 98%. In vivo activity: wound in rats reduced by 95% after 12 days in N/CQDs and N/MC groups.	[26]
*Aspergillus Flavus*, *Aspergillus fumigatus*, *Aspergillus niger*, *Penicillium notatum*, *Mucor circinelloides*	Chitosan nanoparticles (CSNPs)	*In vitro*	MIC, IZD, MFC, % inhibition	CSNPs prepared at pH 4.4 achieved 100% inhibition for all tested fungal isolates; at pH 4.6 achieved 93% inhibition for *M. cirecinelloides* and 70–75% for other isolates.	[25]
*Rhizopus arrhizus*, *Mucor circinelloides*	Amphotericin B lipid nanocrystals (MAT2203)	*In vitro* *In vivo*-mice	MIC	MAT2203 was 5–10 times more effective compared to LAMB and showed similar performance to LAMB in murine invasive mucormycosis models.	[24]
*Aspergillus flavus*, *Subramaniula thielavioides*	Hybrid release system based on amphotericin B (AmB) intercalated in lamellar materials (montmorillonite–MMT and zinc-aluminum layered double hydroxide–ZnAl LDH)	*In vitro*	Drop diffusion assay, simulated wound fluid (SWF)	MMT-AmB and LDH ZnAl-AmB inhibited fungal strain growth, maintaining inhibition zones up to 7 days, and enabled stable sustained AmB release.	[50]
*Rhizopus microsporus*, *Mucor racemosus*, *Syncephalastrum racemosum*	Silver nanoparticles (AgNPs) using *Pseudomonas indica*	*In vitro*	MIC, IZD	MICs were 50, 50, and 100 µg/mL; inhibition zones were 38 mm, 24 mm, 19 mm for *R. microsporus*, *S. racemosum*, and *M. racemosus*, respectively; antioxidant activity without cytotoxicity.	[23]
*Rhizopus delemar*	Polymeric nanoparticles (PLGA-NPs) containing fluconazole + UOSC-13	*In vitro*	MIC	UOSC-13-PLGA-NPs (15 μg/mL) with free fluconazole reduced the MIC_50_ of free fluconazole by an additional 10-fold.	[22]
*Mucor racemosus*, *Rhizopus microsporus*, *Lichtheimia corymbifera*, *Syncephalastrum racemosum*	Trimetallic copper-selenium-zinc oxide nanoparticles (Tri-CSZ NPs) mycosynthesized with *Aspergillus niger*	*In vitro*	MIC, MFC, IZD	*Mucor racemosus* (zone 56 mm; MIC 1.95 µg/mL), *Syncephalastrum racemosum* (52 mm; MIC 3.9 µg/mL), *Rhizopus microsporus* (43 mm; MIC 7.81 µg/mL), *Lichtheimia corymbifera* (25 mm; MIC 62.5 µg/mL).	[34]
*Mucor racemosus*, *Rhizopus microspores*, *Lichtheimia corymbifera*	Eco-friendly nanoemulsions of lemon peel oil (LPO), turmeric oil (TO), and black seed oil (BSO) loaded in nanochitosan (NCh)	*In vitro*	IZD	Chitosan-based nanoemulsions at 1–3% showed inhibition zones ranging from 17 to 23 mm for all strains.	[33]
*Candida albicans*, *C. tropicalis*, *C. krusei*, *C. glabrata*, *Geotrichum candidum*, *Aspergillus niger*, *Mucor circinelloides*	Lipid carrier nanoformulations with fluconazole (NLC-Flu-MTs) and without fluconazole (NLC-Fle-MTs)	*In vitro*	IZD, MIC	Inhibition zone and MIC values were similar for both formulations for all strains, indicating fluconazole synergy with the nanoformulation.	[32]
*Rhizopus arrhizus*	Polyethyleneimine-functionalized silver nanoparticles (PEI-f-Ag-NPs)	*In vitro*	MIC	MICs of PEI-f-AgNP-1 and PEI-f-AgNP-2 (1.65 and 6.50 μg/mL, respectively) were size- and zeta-potential-dependent; germination inhibition 97.33% and 94% in 24 h.	[31]
*Rhizopus microsporous*, *Syncephalastrum racemosum*	Silica nanoemulsion loaded with bis-sulfonyl compound + LIPDI	*In vitro*	IZD, MIC, CFU	SBDMP under red light achieved up to 47.5% fungal eradication and reduced MIC up to 8-fold compared to dark formulation.	[30]
*Aspergillus flavus*, *A. fumigatus*, *A. niger*, *Penicillium notatum*, *Mucor* sp.	Multifunctional ternary Zn–Co–Fe LDH	*In vitro*	MFC, MIC, disk diffusion	MIC for LDH against *Mucor* sp. and *Penicillium notatum* strains was 33.3 and 61 µg/mL, 125 µg/mL for *A. niger* and *A. flavus* (980 µg/mL); *Penicillium* and *Mucor* showed strong antifungal inhibition (85% and 68.3%).	[37]
*Aspergillus flavus*, *A. fumigatus*, *A. niger*, *Candida albicans*, *Mucor rhizopus*, *Penicillium notatum*	Sodium titanate nanotubes (NaTNTs)	*In vitro * * In vivo-*rat	CFM, MIC, disk diffusion	NaTNT showed up to 98% fungal inhibition in vitro and 95% reduction in wound area in vivo, confirming potent antifungal effect and accelerated healing.	[29]
*Rhizopus arrhizus*	Nanoliposomal amphotericin B (NLAmB)	*In vitro*	MIC	NLAmB showed the best antifungal results against *Rhizopus arrhizus*, MIC_50_ 0.063 µg/mL, MIC_90_ 0.25 µg/mL compared to LAMB.	[28]
*Cryptococcus neoformans*	PEGylated dectin-targeted immunoliposome (Dec2/Dec3-AmB-LLs) containing AmB	*In vivo*-rat	-	In vivo efficacy: Dec2/Dec3-AmB-LLs matched AmB-LLs at high pulmonary dose, exceeded AmB-LLs at intermediate dose (1.5 mg/kg) in lungs, and Dec2-AmB-LLs were superior systemically (fewer CFU in all organs and longer survival).	[39]
*Aspergillus flavus*, *A. fumigatus*, *A. niger*, *C. albicans*, *M. indicus*, *Penicillium notatum*	Quinary layered double hydroxide Zr Al Fe Co Ni and its quaternary (Al Fe Co Ni) and tertiary (Fe Co Ni) derivatives	*In vitro*	MIC, CFM, anti-biofilm, % inhibition	In vitro antifungal activity significant: ZrAlFeCoNi most active (MIC = 21 µg/mL; MFC = 16 µg/mL; inhibition zone = 33 mm; 97% inhibition), mainly against *Mucor indicus.*	[27]
*Fusarium solani*, *Aspergillus flavus*, *A. fumigates*, *A. niger and Mucor* spp.	Novel bioinspired lead oxide (PbO) and iron oxide (Fe_2_O_3_) nanoparticles mediated by *Papaver somniferum* L.	*In vitro*	IZD	PbO and Fe_2_O_3_ nanoparticles showed significant antifungal activity, mainly against *F. solani*, with inhibition zones up to 19 mm.	[21]
*Mucor* sp., *Rhizopus* sp., *Candida albicans*, *Penicillium notatum*, *Aspergillus flavus*, *A. fumigatus*, *A. niger*	Ni–Fe LDH nanocomposite loaded/intercalated with DSP	*In vitro*	MIC, CFM, IZD	In vitro antifungal activity significant for tested biomaterials; Ni-Fe LDH/(DSP) nanocomposite MIC = 30 µg/mL, 98% inhibition against *Mucor* sp.	[20]
*Aspergillus niger*, *A. flavus*, *Penicillium commune*, *P. digitatum*	Silver nanoparticles synthesized by *Planococcus maritimus* MBP-2	*In vitro*	IZD	AgNPs showed potent in vitro antifungal activity, especially against *Aspergillus* spp., inhibition zones 4.66–14.33 mm.	[36]
*Fusarium solani*, *Aspergillus fumigatus*, *A. flavus*, *A. niger and Mucor* spp.	N- and S-doped nanocomposites (i.e., FeO/NiO/N-GO and FeO/NiO/S-GO)	*In vitro*	Disk diffusion	Inhibition zones for FeO/NiO, FeO/NiO/GO, FeO/NiO/N-GO, and FeO/NiO/S-GO composites were 7–9 mm; Amphotericin B reference 9–12 mm.	[19]
*Sporothrix brasiliensis*	Microemulsion containing clotrimazole and itraconazole	*In vitro*	IZD	ITC/CLT-ME inhibition zone averaged 43.67 ± 2.31 mm; no skin irritation in mice.	[49]
*Sporothrix* spp.	Keggin-type heteropolyacid silver salts	*In vitro*	MIC, broth kinetics	Ag-HPA salts strongly inhibited *Sporothrix* spp. growth (up to 93%), especially Ag_3_[PMo_12_O_40_] and Ag_3_[PW_12_O_40_]; MICs 8–128 μg/mL; combination with ITC and AMB showed synergistic effect.	[48]
*Candida albicans*, *Sporothrix schenckii*	Luliconazole nanogel based on solid lipid nanoparticles (SLNs) guided by QbD	*In vitro*	IZD	Inhibition zones (ZGI) for SLNG5 (24.67 ± 1.06 and 36.83 ± 0.98 mm) and BM (27.50 ± 0.94 and 33.84 ± 2.20 mm) against *C. albicans* and *S. schenckii*, respectively.	[42]
*Sporothrix schenckii*, *S.brasiliensis*	Chitosan-silver nanocomposites (AgNPs@Chi)	*In vitro* *In vivo*	MIC	Treatment with AgNPs inhibited ≥90% growth of *S. brasiliensis* and *S. schenckii* at 0.12 and 0.25 μg/mL; in vivo tissue regeneration and survival increased to 70% with AgNPs@Chi, vs. 50% with AgNPs.	[44]
*Sporothrix schenckii*, *Candida albicans*	Copper(I) iodide (CuI) nanomaterials (NMs)	*In vitro*	MIC, CFM, drop test/drip dilution	CuI@Ch most effective: *S. schenckii* MIC 12.5 µg/mL, MFC 25 µg/mL (5 h), total inhibition at 75–125 µg/mL; *C. albicans* more resistant, strong reduction after 5 h (≈50 CFU at 12.5 µg/mL; ≈5 CFU at 25 µg/mL); CuI@Ch inhibited with variable performance; isolated Cu NPs less effective.	[47]
*Aspergillus aculeatus*, *A. flavus*, *A. fumigatus*, *A, niger*, *Cryptococcus neoformans*, *Phialophora verrucosa*, *Sporothrix schenckii.*	Plant-mediated biosynthesized silver nanoparticles (AgNPs)	*In vitro*	IZD	Crown flower (*Calotropis gigantea*) extracts inhibited *P. verrucosa* (14.5 mm), *S. schenckii* (13.5 mm), *A. aculeatus* (11.5 mm), *A. flavus* (11 mm), *C. neoformans* (10.5 mm), *A. niger* (9 mm), *A. fumigatus* (9 mm); plant-mediated AgNPs showed zones 13, 9.5, 11, 7.5, 9, 9.5, 10.5 mm, respectively.	[5]
*Candida albicans*, *C. auris*, *Sporothrix schenkii*, *S. brasiliensis*	Clove oil-based nanoemulsion containing amphotericin B	*In vitro*	Disk diffusion, MIC, Checkerboard	NEMLB-05 nanoemulsion inhibition zones: 2.17 cm (*C. auris*), 1.83 cm (*C. albicans*); potent action against *S. brasiliensis* and *S. schenckii*; low IC_50_ (0.0117, 0.0165, 0.0090 mg/mL); up to 3.8× more effective than Amphotericin B; synergy with clove oil (FICI = 0.462).	[45]
*Sporothrix shenckii*	Cocleate containing detoxified LPS (AFCo3-AmB)	*In vitro* *In vivo*-mice *Ex vivo*	MIC, MFC, MABA, CFU of liver and spleen, intracellular activity	AFCo3-AmB and AmB MICs: 0.25 and 1 μg/mL; MFCs: 0.5 and 2 μg/mL; AFCo3-AmB significantly reduced fungal load in spleen and liver at 5 mg/kg for 5 days and eliminated intracellular *S. schenckii* at 0.12 μg/mL (AmB: 0.5 μg/mL).	[46]
*Candida albicans*, *C. krusei*, *C. parapsilosis*, *Cryptococcus neoformans*, *Sporothrix schenckii*, *S. brasiliensis*, *S. globosa*.	Nano- and microparticles loaded with nitric oxide	*In vitro*	Planktonic viability, biofilm	Nitric oxide-releasing NPs showed potent antifungal action, reducing *C. albicans* viability to 4.9–2.4% and eliminating 100% of biofilm cells; eradicated *C. krusei*, *C. parapsilosis*, *C. neoformans*; *S. globosa* fully eliminated, *S. schenckii* reduced/eliminated, *S. brasiliensis* susceptible at 10 mg/mL.	[38]
*Sporothrix brasiliensis*, *Candida albicans*	Topical lipid nanoparticles containing itraconazole	*In vitro* *In vivo*: *Galleria mellonella*	MIC, CFM	Itraconazole-loaded NLC maintained antifungal efficacy: *S. brasiliensis* MIC 0.25 μg/mL, MFC 32 μg/mL; *C. albicans* MIC 1 μg/mL, MFC > 128 μg/mL; free itraconazole MIC 0.12 μg/mL; in vivo 20–40 mg/kg NLC-ITC increased survival of infected larvae from 80% to 100%, ~100% survival for *C. albicans*; free drug ineffective.	[43]
*Candida krusei*, *Tricophyton interdigitale*, *Fonsecaea pedrosoi*	Ethanol extract of the plant *Terminalia fagifolia* and its aqueous fraction used for green synthesis of silver nanoparticles (AgNPs)	*In vitro*	MIC	AgNPs showed strong antifungal activity, MICs 0.10–6.75 μgAg/mL; most sensitive: *C. krusei* (0.10–0.21 µg/mL), followed by *T. interdigitale* (1.69 µg/mL) and *F. pedrosoi* (6.75 µg/mL).	[51]

Species of secondary but still meaningful importance were also included. *Fusarium solani* and *F. oxysporum* were assessed as models of opportunistic and phytopathogenic infections, while *Trichophyton interdigitale* represented dermatophytes involved in cutaneous mycoses [19,21,47,51]. Dematiaceous fungi associated with chromoblastomycosis and phaeohyphomycosis, such as *Fonsecaea pedrosoi* and *Phialophora verrucosa*, appeared less frequently but broaden the investigated spectrum of activity [51].

In addition to the major groups, a few less frequent yet clinically relevant species were also included in the studies, such as *Subramaniula thielavioides*, a dematiaceous fungus associated with eumycetoma and known for its high therapeutic refractoriness. Its inclusion allowed the evaluation of nanocarrier efficacy in infection scenarios that are notoriously difficult to treat [23,30,34].

Finally, *Phialophora verrucosa*, an agent of phaeohyphomycosis and chromoblastomycosis, was employed in specific experimental approaches, contributing to the assessment of antifungal activity against pigmented fungi characterized by chronic evolution and limited therapeutic options [5].

Overall, the comparative analysis of the evaluated studies indicates that fungal responses to nanosystems are strongly influenced by the structural and physiological characteristics of each group [5,19,20,21,22,23,24,25,26,27,28,29,30,31,32,33,34,35,36,37,38,39,40,41,42,43,44,45,46,47,48,49,51]. Fast-growing filamentous fungi with thick, chitin-rich cell walls, such as *Mucorales* and *Penicillium* species, generally exhibit greater susceptibility to metallic nanosystems, which act predominantly through membrane disruption and the induction of oxidative stress [19,20,21,22,23,24,25,26,27,28,29,30,31,32,33,34,35,36,37]. In contrast, encapsulated or melanized fungi, including *Cryptococcus neoformans*, *Sporothrix* spp., and dematiaceous fungi associated with chromoblastomycosis and phaeohyphomycosis, tend to respond more favorably to lipid-based or polymeric nanosystems, which enhance drug penetration, enable controlled release, and reduce host toxicity [5,38,39,40,41,42,43,44,45,46,47,48,49].

## 4. Antifungal Agents and Bioactive Compounds Incorporated into the Nanosystems

**Figure 1:**
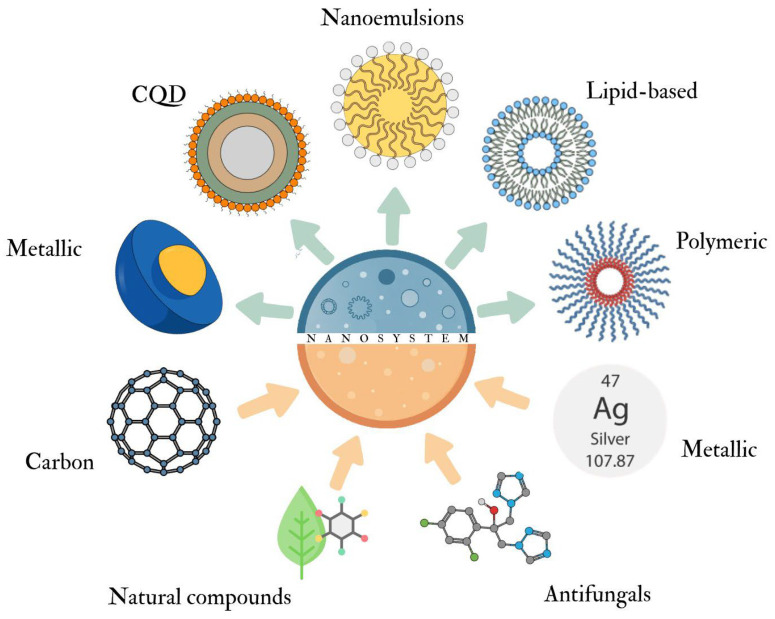
Schematic representation of the main types of nanodrugs (blue arrows) and the antifungal or bioactive compounds incorporated into nanosystems (orange arrows). CQD: Carbon quantum dot.

Various drugs, natural compounds, and metallic or inorganic nanomaterials can be incorporated into nanosystems, forming platforms that enhance the activity of the active agent. Figure 1 provides a schematic overview of the main nanosystem classes and incorporated antifungal agents discussed in this review.

### 4.1. Encapsulation of Classical Antifungal Agents

The encapsulation of classical antifungal agents into nanosystems represents a relevant strategy to overcome therapeutic limitations and antifungal resistance, as these platforms may act through non-classical mechanisms including fungal membrane disruption, generation of reactive oxygen species, interference with biofilm formation, and modulation of the host immune response, thereby enhancing the efficacy of established drugs such as amphotericin B and azoles even against less susceptible strains [52].

Amphotericin B (AmB) was widely employed, both in pegylated liposomal systems (Dec2-AmB-LLs and Dec3-AmB-LLs) featuring fungal targeting through dectin domains, and in classical liposomes (NLAmB) or lipid-based carriers designed for controlled release [28,39,45,46]. Innovative strategies involved the immobilization of AmB in lamellar clays such as montmorillonite or layered double hydroxide, aiming to improve stability and reduce oxidation [50].

Other triazoles were also widely incorporated, such as itraconazole (ITZ), which was encapsulated in lipid-based nanocarriers designed for topical applications, and combined with clotrimazole (CLT) at a 1:1 ratio in nanoformulations to broaden the antifungal spectrum and explore potential synergism [43,49]. Drugs such as fluconazole (FLC), miconazole (MCZ), ketoconazole (KTC), and terbinafine (TBF) were employed in combination with silver salts of heteropolyacids (Ag-HPAs), resulting in enhanced potency and synergistic activity [48]. In other approaches, fluconazole was co-delivered with natural monoterpenes to restore its efficacy against resistant strains, or combined with the synthetic compound UOSC-13 to inhibit melanogenesis and enhance fungal permeability [22,32].

The triazole luliconazole (LCZ) was also incorporated into solid lipid nanoparticles and subsequently formulated into a polymeric gel, with the aim of prolonging drug release and improving cutaneous penetration [42]. In addition, the MAT2203 formulation, containing AmB encapsulated in lipid nanocrystals for oral administration, demonstrated potential to replace the conventional intravenous route while maintaining antifungal activity and reducing toxicity [24].

### 4.2. Natural Compounds and Bioactive Metabolites

Essential oils, such as clove oil (*Syzygium aromaticum*), which is rich in eugenol, as well as turmeric, lemon peel, and *Nigella* sativa seed oils, were incorporated into nanoemulsions and acted synergistically with the nanocarriers by promoting fungal membrane disruption and exerting anti-inflammatory effects [33,45]. Plant extracts rich in polyphenols, flavonoids, and tannins were also employed both as reducing and stabilizing agents and as active components. Examples include flower extracts of *Calotropis gigantea*, containing bioactive cardenolides and pregnanes, and phenolic extracts of *Terminalia fagifolia*, which are rich in ellagic acid derivatives [5,51].

Other innovative compounds included the controlled release of nitric oxide (NO) from S-nitrosothiols, aiming to provide antimicrobial and antibiofilm activity, as well as the use of detoxified lipopolysaccharide (LPS) derived from *Neisseria meningitidis* as an immunomodulatory component in cochleates [38,46].

### 4.3. Metallic and Inorganic Nanomaterials with Intrinsic Activity

A growing trend in the analyzed studies was the use of nanomaterials with intrinsic antifungal activity, eliminating the need for conventional drugs. Multiple formulations employed metallic nanoparticles or metal oxides as active agents, exploring physicochemical mechanisms such as the generation of reactive oxygen species (ROS), ionic release, and electrostatic interactions with the fungal cell wall.

Examples include silver nanoparticles (AgNPs) obtained through biogenic synthesis routes [23,36,44], bimetallic metal oxides (FeO/NiO) and doped graphene derivatives [19], polymer-stabilized CuI [47], and trimetallic CuO–Se–ZnO nanocomposites exhibiting synergistic effects [34]. Other approaches explored multimetallic layered double hydroxides [20,27,37], sodium titanates (NaTNT) [29] or PbO and Fe_2_O_3_ nanoparticles [21].

Biomolecule-functionalized nanomaterials were also developed to enhance biocompatibility and cell–nanoparticle interactions, as in the case of ZnO functionalized with amino acids and surfactants, and AgNPs combined with chitosan to improve stability and modulate release [36,44]. In some formulations, nickel and iron acted in combination with natural polyphenols extracted from date seeds, adding antioxidant and antimicrobial properties. In addition to these examples, PEI-functionalized AgNPs (PEI-f-AgNPs) were also investigated, enabling modulation of surface charge and interaction with the fungal cell wall [31].

Importantly, these strategies are not limited to conventional fungal pathogens but also show promise against emerging multidrug-resistant fungi such as *Candida auris*, which is associated with high mortality, widespread resistance to azoles, polyenes, and echinocandins, and the ability to form persistent biofilms that reduce the efficacy of conventional treatments [53].

### 4.4. Photodynamic Strategies and Emerging Platforms

Some studies investigated alternative approaches to the direct use of antifungal agents, exploring photoinduced and immunochemical mechanisms. These included the use of an organic bis-sulfone photosensitizer (SBDMP) encapsulated in silica nanoemulsions to generate ROS under red-light activation, causing oxidative damage to the fungal cell wall [30]. Other studies employed nitrogen-doped carbon quantum dots and functionalized mesoporous carbon (N/CQDs and N/MC) as intrinsic antifungal agents [26], while chitosan was explored as a natural active agent due to its cationic charge and interaction with the fungal cell wall [25].

## 5. Types of Nanodrugs and Physicotechnical Characteristics

Various substances can be incorporated into nanodrug platforms, including metallic, inorganic, lipid-based, and polymeric materials (Figure 1), forming different types of nanodrugs that facilitate the action of the active agent.

### 5.1. Metallic Nanoparticles and Inorganic Oxides

Metallic nanoparticles represent one of the most prominent categories in the reviewed studies, with AgNPs, ZnO, Fe_3_O_4_, CuO, SeO, CeO_2_, PbO, and Fe_2_O_3_ being the most frequently investigated. They typically exhibit sizes ranging from 10 to 100 nm, predominantly spherical morphology, and a relatively homogeneous distribution (PDI < 0.3), parameters that favor interaction with the fungal cell wall and the generation of reactive oxygen species [19,20,21,23,29,30,31,34,36,37,47].

The incorporation of dopants or surface functionalization was employed in various strategies to modulate physicochemical properties and antifungal activity. For example, PEI-functionalized AgNPs exhibited an increased positive charge and higher affinity for anionic structures of the fungal cell wall [31]. Bimetallic systems, such as trimetallic CuO–Se–ZnO composites [34], exhibited synergism in electrical conductivity, stability, and specific surface area. Meanwhile, metal oxides structured as nanotubes, such as NaTNT, enhanced drug adsorption and controlled release capacity due to their high porosity and anisotropic structure [29].

### 5.2. Lipid Systems and Colloidal Carriers

Lipid-based systems represented another extensively explored class due to their high biocompatibility and ability to encapsulate hydrophobic drugs, including liposomes, nanostructured lipid carriers (NLCs), solid lipid nanoparticles (SLNs), and lipid nanocrystals. Particle sizes ranged from 80 to 250 nm with PDI frequently below 0.25, indicating narrow and homogeneous distribution, and zeta potential values varied from −15 to −35 mV, suitable for aqueous stability and efficient interaction with biological membranes [24,28,39,42,43,45,46,49].

Pegylated liposomes, such as Dec2-AmB-LLs and Dec3-AmB-LLs, exhibited prolonged release and reduced renal toxicity by incorporating polyethylene glycol chains on their surface [39]. AmB lipid nanocrystals (MAT2203) were designed for oral administration, exhibiting high gastrointestinal stability and enhanced bioavailability. In topical systems, SLNs and NLCs containing itraconazole, clotrimazole, or luliconazole demonstrated high encapsulation efficiency (>80%) and sustained release kinetics for up to 48 h [42,43,49].

### 5.3. Polymeric and Hybrid Nanosystems

Polymeric nanosystems were employed both as active carriers and as controlled-release platforms, with emphasis on chitosan, polyethylenimine (PEI), polyethylene glycol (PEG), and oxidized cellulose, materials that provided high structural versatility by allowing fine-tuning of particle size (generally between 100 and 300 nm), modulation of zeta potential, and control over release kinetics [31,38,44,46,51]. Chitosan, in particular, provided a positive surface charge and high affinity for the fungal cell wall, as well as film-forming and biodegradable properties [25,44].

Some innovative systems also incorporated reactive polymeric matrices capable of releasing bioactive species, such as S-nitrosothiols, which controlled nitric oxide release and directly influenced colloidal stability and the diffusion kinetics of the active agent [38]. Similarly, silver heteropolyacid complexes (Ag-HPAs) exhibited a stable nanohybrid structure, with an average size below 150 nm and a negative zeta potential, promoting dispersion in aqueous media and enhancing controlled-release capability [48].

### 5.4. Nanoemulsions and Dispersed Colloidal Systems

Nanoemulsions were widely used as delivery systems for hydrophobic antifungals and natural compounds, notable for their high thermodynamic stability, small droplet size (<200 nm), PDI below 0.3, pseudoplastic behavior, and controlled release kinetics, as well as enabling the co-solubilization of drugs with different polarities [5,33,45,49]. Nanoemulsion systems functionalized with essential oils and plant extracts exhibited additional properties, such as enhanced membrane permeability and antioxidant activity [5,33].

In some cases, the incorporation of biodegradable polymers in the aqueous phase contributed to reducing interfacial tension and enhancing kinetic stability, while the structural versatility of nanoemulsions allowed their adaptation to different administration routes, including topical and mucosal, without the need for significant structural modifications [45].

### 5.5. Photosensitive Platforms and Emerging Nanosystems

An innovative approach involves photosensitive platforms and light- or pH-responsive systems capable of modulating active-agent release and enhancing antifungal effects. Among these are nitrogen-doped carbon quantum dots (N-CQDs) and functionalized mesoporous carbon (N/MC), which exhibited photoluminescent properties, sizes below 20 nm, and high surface area (>300 m^2^/g), features that promote ROS generation under irradiation and potentiate antifungal activity [26].

Similarly, nanoemulsions containing the photosensitizer SBDMP demonstrated controlled release and enhanced photodynamic efficiency under red light, resulting in significant oxidative damage to the fungal cell wall [30]. These advances highlight the current trend of designing multifunctional nanosystems capable of integrating direct antifungal activity, controlled release, and responsiveness to external stimuli within a single therapeutic platform.

## 6. Experimental Models and Administration Routes Evaluated

**Figure 2:**
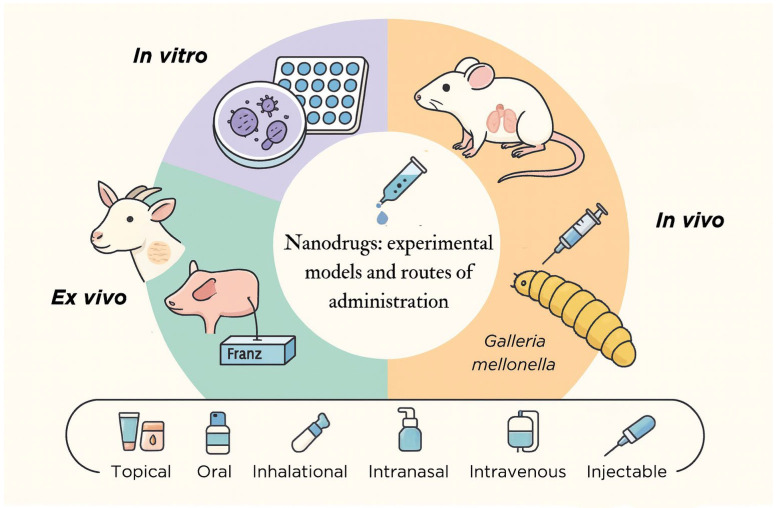
Experimental models and administration routes evaluated using nanodrugs against pathogens that cause subcutaneous mycoses.

Figure 2 summarizes how the studies distributed their assays across different experimental levels and highlights how these approaches contributed to understanding the performance and applicability of antifungal nanosystems.

### 6.1. In Vitro Assays: Initial Approach and Efficacy Screening

Most studies focused on *in vitro* models as an initial evaluation step, employing classical antifungal susceptibility methodologies, including agar diffusion, broth microdilution, determination of minimum inhibitory concentration (MIC), minimum fungicidal concentration (MFC), and radial mycelial growth assays [5,19,20,21,22,23,27,28,30,31,32,33,34,36,37,38,42,45,48,51]. These approaches allowed the preliminary assessment of the antifungal efficacy of the nanosystems across different fungal genera, as well as their activity against both yeast-like and mycelial forms.

In addition to conventional tests, several studies included mechanistic and functional assays, such as membrane integrity evaluation using SYTOX Green [48], confocal and electron microscopy for analysis of internalization and morphological alterations [22,31], intracellular ROS and singlet oxygen assays [30] and studies on biofilm formation and its inhibition [27]. Biocompatibility characterization was also extensively explored, including hemolysis assays [47], cellular cytotoxicity [21,33,36] and interactions with macrophages [46].

In some cases, environmental and physiological variations were simulated to approximate results to real infection conditions, such as assessing the influence of pH on antifungal activity [25] and the analysis of nanoparticle activity under varying osmotic concentrations [37].

### 6.2. Ex Vivo Models: Skin Barriers and Permeation

*Ex vivo* models were employed as an intermediate step between laboratory assays and animal testing, particularly to evaluate skin permeation, deposition, and transdermal release of nanosystems. Pig ear and goat skin were used in Franz diffusion cells to analyze the penetration and distribution of nanostructured formulations, models that, due to their properties similar to human skin, are particularly relevant for the development of topical therapies targeting subcutaneous and cutaneous infections, such as sporotrichosis and eumycetoma [42,43,49].

### 6.3. In Vivo Models: Therapeutic Validation and Translational Relevance

Although less frequent, *in vivo* studies represent the most relevant step for preclinical validation of antifungal nanosystems, allowing the assessment of pharmacological parameters, therapeutic efficacy, and systemic safety; accordingly, various strategies have been explored.

Murine models of pulmonary mucormycosis employed immunosuppressed CD-1 mice infected intratracheally with *Rhizopus arrhizus* or *Mucor circinelloides*, which were subsequently treated with MAT2203 orally and compared to the conventional amphotericin B formulation administered intravenously, with outcomes assessed including survival, fungal burden in the lungs and brain, histopathology, and tolerability [24].

Cryptococcal infection models involved mice infected via intranasal and intravenous routes to evaluate the efficacy of AmB-containing liposomes, administered either intranasally or systemically. The study monitored fungal burden in multiple organs and survival [39].

Subcutaneous sporotrichosis models involved C57BL/6 mice infected with *S.brasiliensis*, which received daily topical treatment with nanosuspensions applied directly to the lesion, compared to systemic itraconazole [44].

Intraperitoneal models of systemic infection involved Balb/c mice infected with *S. schenckii* and treated with AFCo3-AmB via the intraperitoneal route, allowing the assessment of fungal burden reduction in target organs [46].

Alternative infection models employed *Galleria mellonella* larvae to evaluate toxicity, therapeutic efficacy, and impact on survival following fungal infection and treatment with nanosystems [43].

Other in vivo studies investigated cutaneous wounds infected with *Mucor indicus* or *Mucor rhizopus*, assessing wound contraction, fungal burden, and histopathology after topical treatment with nanostructured formulations, models that more accurately reproduce the infectious microenvironment and the behavior of the local tissue response [26,29].

### 6.4. Explored Administration Routes

The proposed administration routes directly reflect the nature of the target infections and the physicochemical characteristics of the developed nanosystems. The topical route was the most frequently investigated, particularly for cutaneous and subcutaneous mycoses, due to its ability to achieve high local concentrations with minimal systemic toxicity. Topical formulations were applied in various formats, including ointments, gels, sprays, emulsions, and polymeric films [5,22,23,29,30,31,32,33,34,37,38,42,43,44,45,46,47,48,49,51].

The oral route was primarily explored with the MAT2203 nanoformulation, which enabled effective administration of AmB in systemic mucormycosis models [24]. The intranasal route was used in pulmonary cryptococcosis models to target treatment directly to the site of infection [39], while the intraperitoneal route was employed in studies of disseminated *S. schenckii* infection [46]. Intravenous administration remained an important comparator in murine models, serving as a control to assess the efficacy and toxicity of nanosystems relative to conventional formulations [24,39,44].

Alternative models included proleg injection in *Galleria mellonella* [43] and inhalation administration proposed for particles with respiratory potential [31,33], they broadened therapeutic perspectives and demonstrated the versatility of nanosystems across different clinical contexts.

## 7. Methods for Evaluating Antifungal Activity

### 7.1. Classical Antifungal Susceptibility Assays

The vast majority of studies employed methods standardized by the Clinical and Laboratory Standards Institute (CLSI), primarily protocols M27-A3 (for yeasts) [54] and M38-A2 (for filamentous fungi) [55], with emphasis on the following assays:

Broth microdilution is used to determine the minimum inhibitory concentration (MIC) and, in many cases, the minimum fungicidal concentration (MFC), defined as the lowest concentrations capable of inhibiting or killing fungal growth [20,21,22,23,27,28,29,32,33,34,37,38,39,43,44,45,46,47,51]. Additional assays included the calculation of MIC_50_ and MIC_90_, geometric means, and cumulative susceptibility distribution [28,44].

Disk and well diffusion is widely applied to measure inhibition zones in millimeters, allowing both qualitative and quantitative assessment of antifungal effects, as well as direct comparisons with conventional antifungals (e.g., AmB, FLC, and ITZ) [5,19,20,21,23,24,25,27,32,33,34,36,42,49,50].

Moreover, some studies combined both methods to strengthen comparative analysis and validate the reproducibility of results, correlating inhibition zone diameters with MIC values obtained by microdilution [30,31,48].

### 7.2. Complementary Functional Assays and Mechanistic Analysis

To deepen the understanding of the mechanisms of action of nanosystems, various studies incorporated functional methodologies and structural analyses.

Sorbitol assays are used to investigate interactions with the cell wall by assessing MIC variations in the presence of an osmotic stabilizer [20,27,29,37].

Membrane permeabilization is analyzed using dyes such as SYTOX Green and propidium iodide (PI), which penetrate only cells with compromised membranes [31,48]. Electron microscopy (TEM and SEM) may reveal morphological changes such as vacuolization, cell wall rupture, cytoplasmic disorganization, and cell lysis, confirming structural damage induced by the nanosystems [31,44,48,51].

Vibrational spectroscopy (SERS) was used to identify damaged biomolecules and metabolic alterations associated with oxidative stress and cellular collapse [31].

Atomic force microscopy (AFM) was used to characterize cell surface topography and reveal changes in adhesion and permeability [47].

The analysis of reactive oxygen species (ROS) and singlet oxygen was also a recurring tool to investigate antifungal mechanisms, where increased ROS production was correlated with metal ion release and the phot [21,30,51].

### 7.3. Synergy Assays and Pharmacological Interaction Studies

The checkerboard assay was widely used to determine the fractional inhibitory concentration index (FICI), allowing interactions to be classified as synergistic (FICI < 0.5), additive, indifferent, or antagonistic [22,44,45]. Combination assays were also employed to compare the antifungal activity of nanosystems in combination with conventional antifungals versus their free forms [32,48].

### 7.4. Biofilm and Virulence Factor Assays

Colony-forming unit (CFU) assays were applied after treatment exposure, along with analyses of residual viability following biofilm disruption [38,47]. In some cases, mycelial melanization was monitored as a virulence marker, correlating pigment inhibition with antifungal efficacy [48]. Assays with phagocytic cells also allowed evaluation of intracellular efficacy against internalized yeasts [46].

### 7.5. Cytotoxicity and Biocompatibility Assays

The safety assessment of nanosystems was integrated with antifungal analysis in several studies. Cell viability assays (MTT and Alamar Blue) were employed to determine their impact on mammalian cell lines and macrophages [33,43,46]. Hemolysis assays complemented the evaluation by measuring compatibility with human erythrocytes [44,47].

Across the studies included in this review, toxicity assessment of nanosystems was heterogeneous and frequently limited to *in vitro* cytotoxicity assays or alternative *in vivo* models. Quantitative IC_50_ values for mammalian cells were inconsistently reported, with several investigations focusing primarily on antifungal efficacy. Nevertheless, when cytotoxicity was assessed, most nanosystems demonstrated acceptable biocompatibility within the therapeutic concentration range, including low hemolytic activity and cell viability above commonly accepted thresholds [33,43,44,46,47]. The lack of standardized toxicological endpoints remains a relevant limitation and highlights the need for harmonized safety evaluation in future studies.

### 7.6. In Vivo Assays and Histopathological Analyses

Parameters measured included mean survival time (MST), survival percentage, logarithmic reduction in fungal burden by qPCR, and CFU quantification per organ [24,39,43,46]. Histological evaluations using Grocott methenamine silver staining revealed the presence and distribution of hyphae in tissues, while immunofluorescence and confocal imaging analyses allowed tracking of liposome and nanoparticle biodistribution in infected organs [39].

## 8. Antifungal Performance and Comparative Quantitative Results

In assays with *Mucorales* spp. and *Penicillium* spp., lamellar and metallic systems exhibited high efficacy and dose-dependent behavior, with Ni–Fe LDH/DSP and NaTNT formulations showing MICs between 15 and 31 µg/mL and equivalent MFCs, achieving up to 98% inhibition for *Mucor* spp. and 83% for *Penicillium* spp., along with inhibition zones exceeding 25 mm and lesion reduction of up to 95% after 12 days [20,29]. The Zn/Co/Fe LDH exhibited MICs of 33–62 µg/mL and inhibition zones of 19–21 mm, attributed to the synergism between metal cations and the formation of reactive oxygen species [37]. The zirconium-containing LDH, Zr–Al–Fe–Co–Ni, was the most potent against the fungal panel, exhibiting an MIC of 21 µg/mL against *Mucor* spp. and achieving up to 97% inhibition [27].

For *Mucor circinelloides*, NLC-Flu-MTs showed a moderate improvement compared to the formulation without fluconazole (MIC 31.2 µg/mL vs. 62.5 µg/mL), while free fluconazole was inactive [32]. Nanocomposites containing Ni and Fe also demonstrated notable performance against *Penicillium digitatum* and *P. commune*, with MICs of 8–16 µg/mL and MFCs around 32 µg/mL, reinforcing their fungicidal profile and concentration-dependent effect [36]. FeO/NiO formulations and their combinations with graphene produced inhibition zones ranging from 6 to 9 mm [19]. Additionally, biosynthesized AgNPs exhibited inhibition halos of 8.66 mm and 4.66 mm against *P. digitatum* and *P. commune*, respectively, in a dose-dependent manner [36].

PEI-f-AgNPs exhibited MICs of 1.65 and 6.50 µg/mL against *Rhizopus arrhizus*, while essential-oil nanoemulsions produced inhibition halos of 17–23 mm against *Mucor racemosus*, *Rhizopus microsporus*, and *Lichtheimia corymbifera*, maintaining physicochemical stability and low cytotoxicity [31,33]. The nanoliposomal AmB (NLAmB) proved to be the most potent agent against 39 *R. arrhizus* isolates, with MIC_50_/_90_ values of 0.063/0.25 µg/mL, outperforming free AmB and the azoles [28].

Nanoemulsions containing neutral polymers showed an MIC of 1.56 µg/mL, an MFC of 3.12 µg/mL, and an IC_50_ of 13 µg/mL, indicating high antifungal potency and cellular safety [34]. The nanoencapsulated fluconazole demonstrated a significant increase in efficacy against *Rhizopus delemar*, with up to an eightfold reduction in MIC compared to the free form, highlighting a clear biopharmaceutical advantage [22]. The nanoencapsulated AmB consistently demonstrates advantages in targeting and potency: in cryptococcosis models, the liposomal formulation decorated with Dectin-2/Dectin-3 (DectiSomes) optimized drug delivery, resulting in approximately a 100-fold reduction in brain fungal burden compared to unmodified liposomal AmB, and increased median survival to 18.5 days [39].

The nanosystems MAT2203 promoted a 1.5–2 log reduction in pulmonary and cerebral fungal burden, with an increase in survival from 9 to 13.5 days in mucormycosis models, showing performance comparable to or superior to liposomal amphotericin B [24]. The N/CQDs and N/MC nanosystems showed MICs of 1.95–62.5 µg/mL and MFCs of 2–8 µg/mL, with complete re-epithelialization observed within 12 days, confirming their topical efficacy and regenerative effect [26].

Chitosan nanoparticles exhibited MICs ranging from 0.125 to 2 mg/mL and inhibition halos of 30–40 mm at pH 5, a condition in which protonation enhances interaction with the fungal cell wall [25]. Lamellar systems containing amphotericin B maintained sustained release for up to 60 days, ensuring prolonged activity and therapeutic stability [50].

In *Sporothrix* spp., metallic and colloidal approaches yielded substantial quantitative results, where AgNPs and AgNPs@Chi demonstrated MIC_50_ values between 0.06 and 0.25 µg/mL and MIC_90_ ≤ 1 µg/mL, with 70% survival *in vitro* an *in vivo* model and complete regression of cutaneous lesions [44]. Biosynthesized AgNPs produced a 9.5 mm inhibition zone against *S. schenckii* [5]. AFCo3-AmB showed an MIC of 0.25 µg/mL and an MFC of 0.5 µg/mL against *S. schenckii* in *in vitro* assays, and significantly reduced fungal burden in *in vivo* models of systemic sporotrichosis [46]. Nanoemulsions also exhibited high activity against *S. brasiliensis* and *S. schenckii*, outperforming fluconazole in some resistant strains, with IC_50_ values of 0.0165 mg/mL and 0.0090 mg/mL, respectively [45]. S-nitrosothiol nitric oxide-releasing nanoparticles (SNO-NPs) completely eradicated *S. globosa* and *S. brasiliensis* (the latter at 10 mg/mL) [38].

Ag-HPA salts exhibited MICs ranging from 8 to 128 µg/mL, 93% inhibition, and inhibition halos exceeding 70 mm when combined with itraconazole and AmB, demonstrating pronounced synergism [48]. The topical formulations—polymeric nanogel and the ITC/CLT-ME microemulsion—achieved inhibition halos of 36.8 ± 0.9 mm and 43.7 ± 2.3 mm, respectively, exhibiting high skin permeability, absence of irritation, and a biocompatible profile [42,49]. The nanoencapsulated itraconazole (NLC) maintained an MIC of 0.25 µg/mL against *S. brasiliensis* and an MFC of 32 µg/mL, increased survival of infected larvae to 100% at lower doses, and minimized transdermal penetration [43].

In other filamentous fungi, CuI@Ch showed an MIC of 12.5 µg/mL, an MFC of 25 µg/mL, and complete elimination within 5 h, with a 98% reduction in cell viability [47]. *Fusarium solani* exhibited inhibition halos of up to 19 mm at 10 mg/mL for PbO nanoparticles, outperforming Fe_2_O_3_ under the same conditions Photodynamic therapy based on functionalized silica nanoemulsions resulted in complete eradication of *Rhizopus microsporus* and *Syncephalastrum racemosum*, associated with intense reactive species generation and hyphal deformation [30]. *Pseudomonas indica*-mediated AgNPs maintained fungicidal activity at 30 µg/mL and an IC_50_ of 132 µg/mL, indicating a broad therapeutic window [23]. AgNPs also demonstrated inhibitory activity against *T. interdigitale* and *F. pedrosoi*, with MICs of 1.69 µgAg/mL and 6.75 µgAg/mL, respectively [51].

## 9. Conclusions

Various nanosystems demonstrate promising potential for the treatment of subcutaneous mycoses, showing confirmed efficacy *in vitro, ex vivo*, and *in vivo*, with the ability to reduce MICs, CFUs, and lesion areas, as well as to enhance wound healing and survival in experimental models. Strategies involving metallic, polymeric, lipid-based nanoparticles, and nanoemulsions have proven capable of overcoming the pharmacological limitations of conventional antifungals, such as low tissue penetration, toxicity, and prolonged treatment requirements. This review highlighted that combining different nanostructured platforms with antifungal drugs or bioactive agents provides additional benefits, including controlled release, targeted delivery, and therapeutic synergism. Despite these promising results, several challenges remain, including potential nanotoxicity, scalability of production, and regulatory approval. Future research should prioritize standardized experimental protocols, systematic in vivo safety and efficacy assessments, pharmacokinetic and pharmacodynamic studies, and the exploration of combined nanoformulations to maximize clinical applicability. Therefore, nanotechnology represents a relevant and growing approach for developing more effective and targeted therapies against subcutaneous mycoses, with the potential to significantly improve the management of these complex infections.

## Data Availability

No new data were created or analyzed in this study.

## Supplementary Materials

The following supporting information can be downloaded at: https://www.mdpi.com/article/10.3390/microorganisms14010187/s1, Table S1. Database search strategies and their respective search results (number of publications).
