# Supplementary material for: Nanodrugs for Subcutaneous Mycoses: Applications, Antifungal Performance, and Translational Perspectives
Source: Microorganisms. 2026 Jan 14;14(1):187. doi: 10.3390/microorganisms14010187 (PMC12844405; doi:10.3390/microorganisms14010187)
Supplement: Supplementary file 1 [file microorganisms-14-00187-s001.zip › microorganisms-4057313-supplementary.pdf]

Table S1. Database search strategies and their respective search results (number of publications).

Database	Boolean Search (Keywords combination)	Result
PubMed<br>Scopus<br>Web of Science<br>Scielo	((Nanotechnology OR Nanotechnologies OR Nanostructure OR Nanostructures OR Nanomaterial OR Nanomaterials OR "Nanostructured Material" OR "Nanostructured Materials" OR "Nanocrystalline Material" OR "Nanocrystalline Materials" OR Nanocrystal OR Nanocrystals OR Nanoparticle OR Nanoparticles OR "Drug Carrier" OR "Drug Carriers" OR nanomedicine OR "Nano Drug Delivery System" OR "Nano Drug Delivery Systems" OR "Nano-Drug Delivery System" OR "Nano-Drug Delivery Systems" OR "Nano Delivery System" OR "Nano Delivery Systems" OR NDDS OR NDDSs OR "Nanoparticle Based Drug Delivery System") AND (*Sporothrix* OR *Sporotrichosis* OR *"Sporothrix schenckii"* OR *Chromoblastomycoses* OR *Chromomycosis* OR *Chromomycoses* OR *"Dermatitis Verrucosa"* OR *Chromoblastomycosis* OR *Lobomycoses* OR *Lobomycosis* OR *"Jorge Lobo Disease"* OR *"Jorge Lobos Disease"* OR *"Keloidal Blastomycosis"* OR *"Keloidal Blastomycoses"* OR *Lacaziosis* OR *Lacazioses* OR *"Lacazia loboi Infection"* OR *"Lacazia loboi Infections"* OR *"Lobomycosis like Disease"* OR *"Lacaziosis like Disease"* OR *Maduromycosis* OR *Eumycetoma* OR *Actinomycetoma* OR *"Madura Foot"* OR *Mycetoma* OR *Phaeohyphomycoses* OR *"Subcutaneous Phaeohyphomycosis"* OR *"Subcutaneous Phaeohyphomycoses"* OR *Phaeohyphomycosis* OR *Rhinosporidioses* OR *"Rhinosporidium seeberi infection"* OR *"Rhinosporidium seeberi infections"* OR *Rhinosporidiosis* OR *"entomophthorales infection"* OR *"Conidiobolus coronatus infection"* OR *basidiobolomycosis* OR *rhinoconidiobolomycosis* OR *rhinoentomophthoramycosis* *"Conidiobolus infection"* OR *"conidiobolomycosis"* OR *"Basidiobolus ranarum infection"* OR *entomophthoromycosis* OR *Zygomycoses* OR *Phycomycosis* OR *Phycomycoses* OR *Entomophthoramycosis* OR *Entomophthoramycoses* OR *Zygomycosis* OR *"Mucorales Infections"* OR *Mucormycoses* OR *Mucormycose* OR *"Mucorales Infection"* OR *Mucormycosis))*	201
Science Direct	((Nanotechnology OR Nanoparticle OR "Drug Carrier" OR nanomedicine OR "Nano Drug") AND (*Sporotrichosis* OR *Chromoblastomycosis* OR *Lobomycosis* OR *Eumycetoma* OR *Phaeohyphomycosis* OR *Rhinosporidiosis* OR *entomophthoromycosis* OR *Mucormycosis*))	460

The keyword search strategy in this database differed from that used in the other database due to word/character limitations in the search field.
